# Targeting TMEM88 as an Attractive Therapeutic Strategy in Malignant Tumors

**DOI:** 10.3389/fonc.2022.906372

**Published:** 2022-06-06

**Authors:** Ming Cai, Wei-Jian Ni, Ying-Hong Wang, Jing-Ji Wang, Hong Zhou

**Affiliations:** ^1^ Department of Pharmacy, The Second Affiliated Hospital of Anhui University of Chinese Medicine, Hefei, China; ^2^ Anhui Acupuncture and Moxibustion Clinical Medicine Research Center, The Second Affiliated Hospital of Anhui University of Chinese Medicine, Hefei, China; ^3^ Inflammation and Immune Mediated Diseases Laboratory of Anhui Province, The Key Laboratory of Anti-inflammatory of Immune Medicines, Ministry of Education, Anhui Institute of Innovative Drugs, School of Pharmacy, Anhui Medical University, Hefei, China; ^4^ Anhui Provincial Hospital, The First Affiliated Hospital of USTC, Division of Life Sciences and Medicine, University of Science and Technology of China, Hefei, China; ^5^ Department of Pharmacy, Anhui Provincial Cancer Hospital, The First Affiliated Hospital of USTC, Division of Life Sciences and Medicine, University of Science and Technology of China, Hefei, China

**Keywords:** malignant tumor, transmembrane protein 88 (Tmem88), Wnt/β-catenin, target, therapeutic strategy

## Abstract

According to authoritative surveys, the overall morbidity and mortality of malignant tumors show an upward trend, and it is predicted that this trend will not be well contained in the upcoming new period. Since the influencing factors, pathogenesis, and progression characteristics of malignant tumors have not been fully elucidated, the existing treatment strategies, mainly including surgical resection, ablation therapy and chemotherapy, cannot achieve satisfactory results. Therefore, exploring potential therapeutic targets and clarifying their functions and mechanisms in continuous research and practice will provide new ideas and possibilities for the treatment of malignant tumors. Recently, a double-transmembrane protein named transmembrane protein 88 (TMEM88) was reported to regulate changes in downstream effectors by mediating different signaling pathways and was confirmed to be widely involved in cell proliferation, differentiation, apoptosis and tumor progression. At present, abnormal changes in TMEM88 have been found in breast cancer, ovarian cancer, lung cancer, thyroid cancer and other malignant tumors, which has also attracted the attention of tumor research and attempted to clarify its function and mechanism. However, due to the lack of systematic generalization, comprehensive and detailed research results have not been comprehensively summarized. In view of this, this article will describe in detail the changes in TMEM88 in the occurrence and development of malignant tumors, comprehensively summarize the corresponding molecular mechanisms, and explore the potential of targeting TMEM88 in the treatment of malignant tumors to provide valuable candidate targets and promising intervention strategies for the diagnosis and cure of malignant tumors.

## Introduction

**Figure 1:**
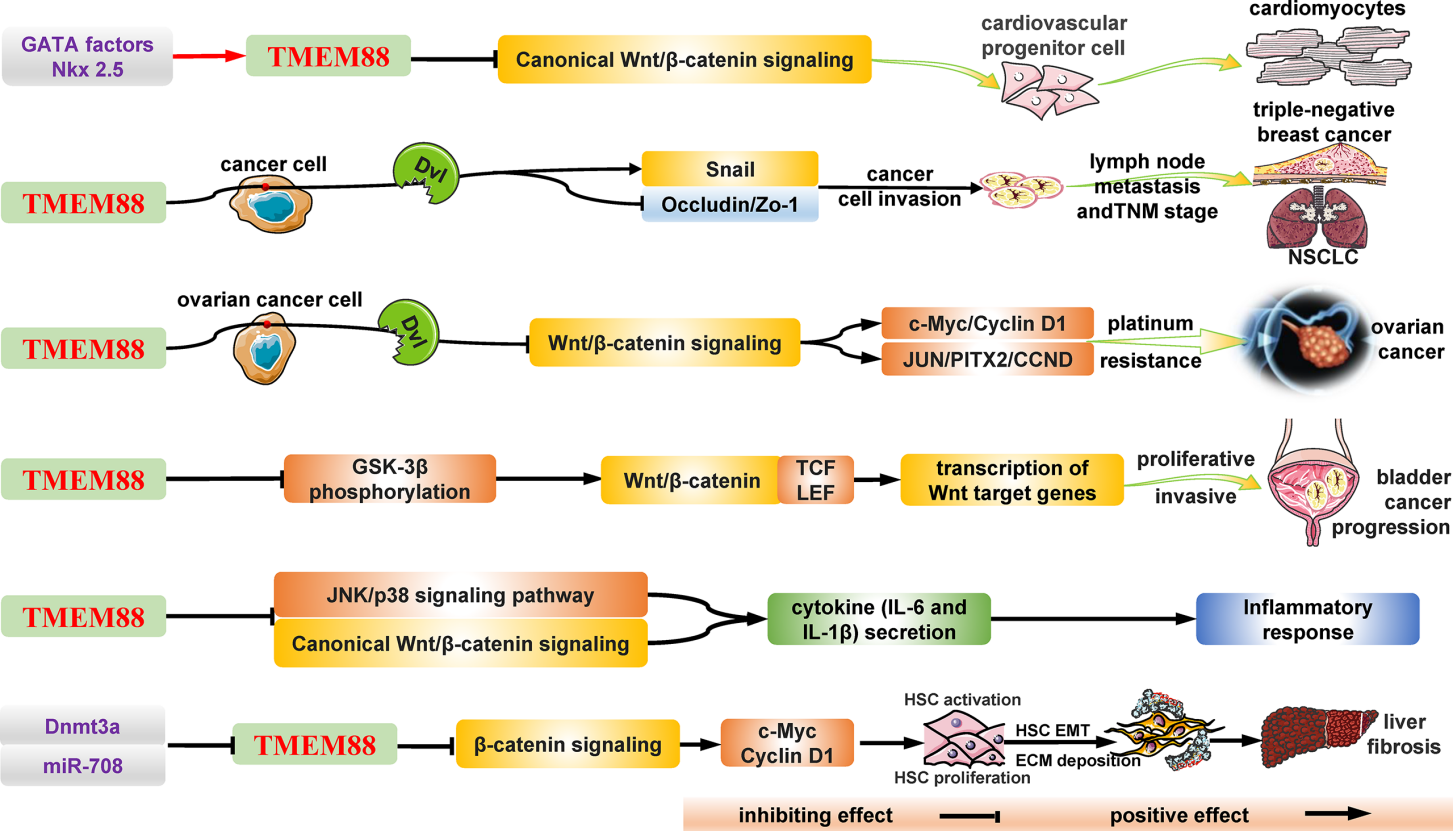
The structure and general biological functions of TMEM88 in humans. As a secondary transmembrane protein, TMEM88 is widely distributed in many types of cells and tissues and plays an important regulatory role in various diseases and pathological processes, such as cancers, fibrosis, and inflammatory responses. Under stimulation, including DNA methylation, noncoding RNA and the inflammatory environment, the mRNA and protein levels of TMEM88 changed significantly. Subsequently, the significantly changed TMEM88 regulates the classical and noncanonical Wnt/β-catenin signaling pathways through different mechanisms in the cytoplasm and nucleus, such as the interaction with DVL proteins, thereby affecting the transcription and expression of downstream target genes. Ultimately, these effector target molecules affect the growth and development of tissues and organs and the progression of diseases, such as tumors by regulating various cellular life activities, such as cell proliferation, migration, invasion, and drug resistance. CCND, Cyclin D1; c-Myc, c-Myc proto-oncogene; Dnmt3a, DNA (cytosine-5)-methyltransferase 3a; DVL, PDZ domain of Dishevelled-1; ECM, extracellular matrix; EMT, epithelial-mesenchymal transition; GATA, GATA transcription factor; GSK-3β, glycogen synthase kinase-3β; HSC, hepatic stellate cell; JNK, c-Jun N-terminal kinase; JUN, jun proto-oncogene; NSCLC, non-small-cell lung cancer; PITX2, paired-like homeodomain 2; TCF/LEF, T-cell factor/lymphoid enhancer-binding factor; TMEM88, transmembrane protein 88; TNM, tumor node metastasis.

In recent years, membrane proteins have been studied and have become a research hotspot because they are widely distributed in tissues and play important and complex roles in various physiological processes and multiple diseases. Authoritative studies have shown that membrane proteins are distributed in the membranes of various cells and organelles, accounting for approximately 25% of the human proteome (1, 2), and exert undeniable effects on signal transduction in the cell-external environment and cell-cell interactions (3). Several studies have pointed out that membrane proteins can be classified into peripheral membrane proteins, lipid-anchored membrane proteins and integral membrane proteins according to their own structure and how they bind to biological membranes. The integral membrane protein contains at least one transmembrane fragment, which is also a transmembrane protein. These transmembrane proteins are not only involved in the body’s metabolism and functional regulation (4–7) but also perform considerable regulatory roles in the tumorigenesis and progression of several tumors (8–11). For example, ATG9A, the only transmembrane protein currently in the core machinery of autophagy, can affect pathophysiological events such as cell growth, proliferation, stress and injury by regulating autophagy (12). In addition, endoplasmic reticulum-associated transmembrane protein 166 (TMEM166) was found not only in a variety of normal tissues and organs but also involved in multiple pathological processes, including cancer, infection, neurodegeneration, autoimmune disease, and sexually transmitted diseases, by regulating programmed cell death (13). Moreover, a transmembrane protein called the tyrosine kinase receptor may be abnormally activated to affect tumor cell growth, metastasis, invasion and malignant transformation by regulating multiple subprotein families and downstream signaling pathways (14). The aforementioned findings have made the transmembrane protein family a research focus that has received extensive attention. As an indispensable transmembrane protein family member, transmembrane protein 88 (TMEM88) has also been found to affect the growth and development of the body in normally expressed tissues, such as the development of cardiomyocytes and the activation of hematopoietic stem cells, while abnormal expression can affect the progression of various diseases, such as the inflammatory response, extracellular matrix secretion and drug resistance (15–21) (**Figure 1**). As an important tumor suppressor gene, TMEM88 benefits from the regulation of the Wnt signaling pathway and its downstream target genes and is widely involved in various biological events of malignant tumor cells, which has potential research value (22, 23). However, the current research on TMEM88 is still in its infancy, and there is no complete summary to help researchers fully understand the important role of TMEM88 in tumor research and prevention. In these circumstances, exploring TMEM88 as a new biomarker for tumor diagnosis and confirming that TMEM88 has become a promising target will be the research and prevention of tumors. In view of this, this review comprehensively describes the structure and function of TMEM88 and discusses its progress in the study of malignant tumors to provide important references and directions for subsequent research.

## General Structure and Biological Function of TMEM88

The TMEM88 gene is a plus strand gene consisting of 1214 bases located at the p13.1 position on chromosome 17, and the corresponding TMEM88 protein is a 159 amino acid residue with a molecular weight of 17251 Da. An initial study found that the TMEM88 molecule is expressed on the Xenopus embryonic cell membrane (24). Meanwhile, the study also found that when the expression of TMEM88 was significantly upregulated in human embryonic kidney cells, the downstream Wnt/β-catenin signal transduction process was inhibited, while silencing TMEM88 could activate the Wnt/β-catenin signal transduction process. Another study found that TMEM88 can inhibit Wnt/β-catenin signal activation in human embryonic stem cells to a certain extent, thereby regulating the differentiation and development of embryonic stem cells into cardiomyocytes (21). With the progress of research, it was found that the TMEM88 protein is widely present in a variety of tumors, but its functions are different due to the different tissue and subcellular locations (**Table 1**). One study found that cytoplasmic localization of TMEM88 was positively correlated with TNM stage and lymph node metastasis in triple-negative breast cancer, whereas nuclear localization was negatively correlated with lymph node metastasis in nontriple-negative breast cancer (30); in ovarian cancer, TMEM88 was found to downregulate the levels of c-Myc and Cyclin D1, thereby inhibiting the proliferation of ovarian cancer cells (19). In non-small-cell lung cancer (NSCLC), the high expression of TMEM88 is positively correlated with a better prognosis of patients, but the detailed mechanism needs to be further clarified (28).

**Table 1:** The landscape of the roles of TMEM88 in various human tissues.

Tissue localization	Disease types	Associated tissues or cells	Alterations	Target	Function	References
Human embryo	cell development	Human embryonic stem cells	Upregulation	DVL	Regulate cardiomyocyte specification	Lee, Heejin et al. (22) Lee, Ho-Jin et al. (24) Palpant, Nathan J et al. (21)
Ovarian	ovarian cancer	CP70 and PEO4	Upregulation	JUN, PTIX2, β-catenin, c-Myc and cyclin-D1	Regulate platinum resistance	de Leon, Maria et al. (19)
		A2780 and PEO1	Downregulation			
Thyroid	thyroid cancer	BCPAP, TPC1, K1 and NPA87	Downregulation	TCF/LEF, c-Myc and cyclin D1	Suppress tumor process	Geng, Qianqian et al. (25)
Lung	Lung cancer	A549, H1299, H460, H292, SPC-A-1 and LTEP-A-2	Downregulation	DVL, FZD and ROR1	Suppress tumor process	Zhang, Xiupeng et al. (26) Stewart, David J. (27) Ma, Rongna etal. (28)
Skin	keloids	Keloid fibroblasts	Downregulation	β-catenin, c-Myc and cyclin D1	Inhibit extracellular matrix expression	Zhao, Huafei et al. (16)
Bladder	bladder cancer	5637, UM-UC-3, T24 and SW780	Downregulation	GSK-3β, β-catenin and TCF/LEF	Suppress tumor process	Zhao, Xu et al. (29)
Breast	triple-negative breast cancer	MCF-7, HER18, MDA-MB-231 and MDA-MB-468	Upregulation	DVL, Snail, Occludin and Zo-1	Promote tumor process	Yu, Xinmiao et al. (30)
Liver	liver fibrosis	LX-2 and human liver fibrotic tissues	Downregulation	β-catenin, Wnt3a, Wnt2b, Wnt10b, p-JNK and p-P38	Regulate proinflammatory cytokine secretion and inhibit HSC excitation	Xu, Tao et al. (17) Xu, Tao et al. (31)

c-Myc, c-Myc proto-oncogene; DVL, PDZ domain of Dishevelled-1; FZD, Wnt receptor Frizzled; GSK-3β, glycogen synthase kinase-3β; HSC, hepatic stellate cell; JNK, c-Jun N-terminal kinase; JUN, jun proto-oncogene; >PITX2, paired-like homeodomain 2; ROR1, receptor tyrosine kinase-like orphan receptor 1; TCF/LEF, T-cell factor/lymphoid enhancer-binding factor; TMEM88, transmembrane protein 88.

## Role and Mechanism of TMEM88 in Malignant Tumors

**Figure 2:**
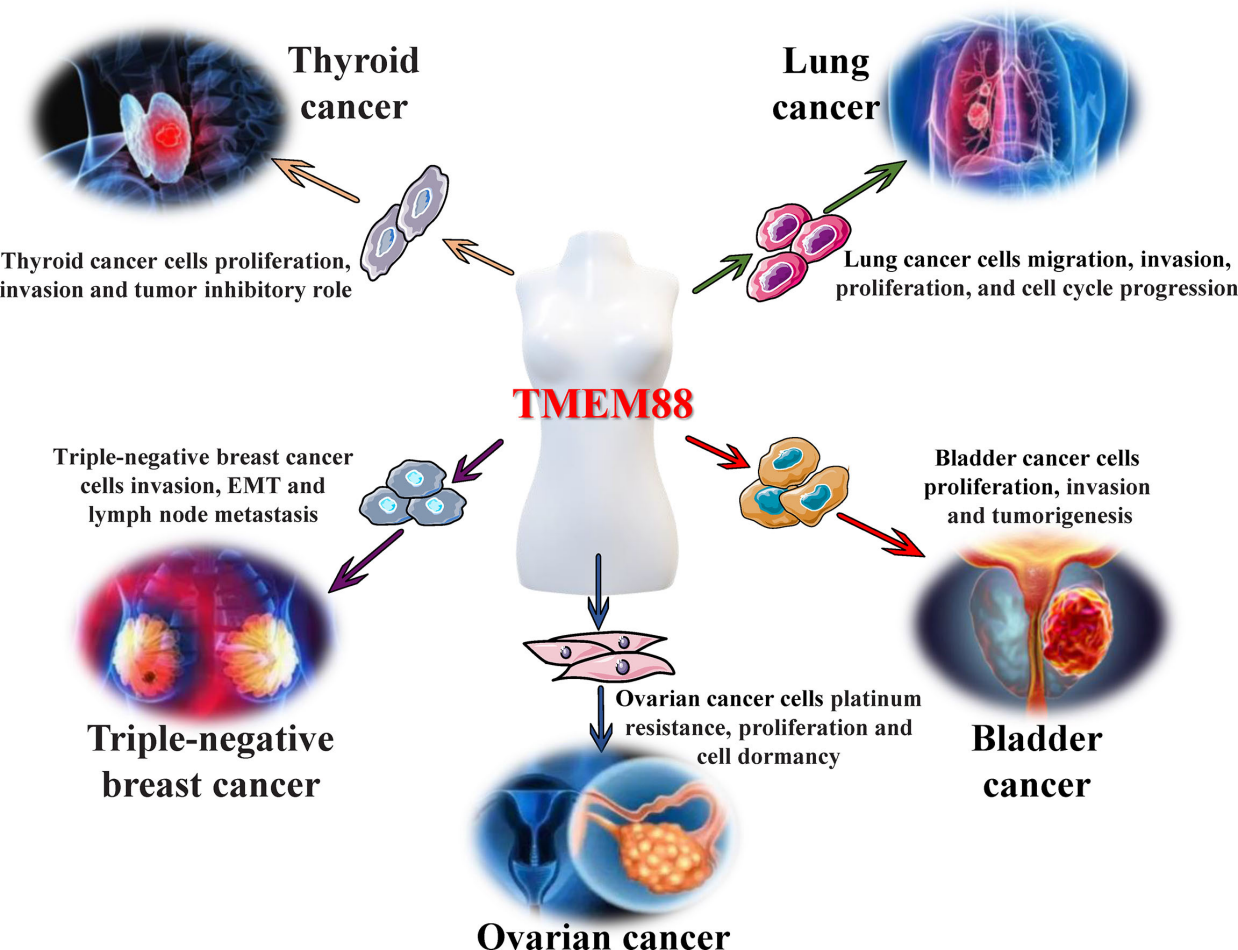
Regulatory effects of TMEM88 in malignant tumors. Different tumor microenvironments, such as inflammation and oxidative stress, can cause significant changes in the levels of TMEM88 in tissues and cells, which will affect tumor cell properties, including abnormal cell proliferation, invasion, migration, and epithelial-mesenchymal transition (EMT), through canonical and noncanonical Wnt/β-catenin signaling and other signal transduction pathways. These tumor cell properties lead to the occurrence, growth, invasion, and metastasis of various tumors, including ovarian cancer, thyroid cancer, triple-negative breast cancer, bladder cancer, and lung cancer, and ultimately affect tumor progression and poor prognosis.

In recent years, an increasing number of research results have shown that abnormal expression of TMEM88 exists in several malignant tumors (ovarian cancer, breast cancer, lung cancer, etc.) and has actively participated in the abnormality of tumor cell proliferation, invasion, metastasis and apoptosis. Meanwhile, with the development of structural biology, biochemistry and other disciplines, breakthroughs and progress have been made in the isolation and characterization of transmembrane proteins, which indicates that it may become a promising molecular marker and valuable intervention target for tumor diagnosis, therapy and prognosis (**Figure 2**).

### TMEM88 in Ovarian Cancer

Ovarian cancer has become a malignant tumor in the female germline that seriously threatens women’s health, with its third incidence rate (after cervical cancer and endometrial cancer) and the highest mortality rate. On the one hand, due to the lack of typical clinical manifestations in the early stage, a large proportion of patients are already in the middle and late stages when they are diagnosed, and even the tumor cells have metastasized far away. On the other hand, platinum-based chemotherapy is the main means to maintain the survival of such patients, but many patients suffer from drug resistance, tumor recurrence and metastasis after chemotherapy (32). Based on this, exploring the pathogenesis and drug resistance mechanism of ovarian cancer and finding effective targets are of great significance for improving treatment and improving the 5-year survival rate of ovarian cancer patients. Maria de Leon et al. used an Illumina 450k DNA methylation array to detect methylated genes and levels in ovarian cancer xenografts and found that compared with platinum-sensitive transplanted tumors, the promoter region of the TMEM88 gene in platinum-resistant transplanted tumors in nude mice was significantly hypomethylated, while TMEM88 mRNA showed a substantial increase (19). Meanwhile, the application of the DNA methyltransferase inhibitor SGI-110 can significantly increase the TMEM88 mRNA level and corresponding protein expression in platinum-sensitive ovarian cancer cells. Both *in vitro*, *in vivo* and clinical studies have shown that the level of TMEM88 is significantly increased in platinum-resistant ovarian cancer xenograft nude mice, platinum-resistant ovarian cancer cells and recurrent ovarian cancer tissue, while knockdown or decrease the expression of TMEM88 can resensitize tumor cells to platinum drugs. Mechanistic studies found that silencing TMEM88 alleviated the inhibition of canonical Wnt/β-catenin signaling by reducing the interaction of the C-terminal VWV (Val-Trp-Val) sequence with the PDZ domain of Dishevelled-1 (Dvl-1) (19, 23). The activation of Wnt/β-catenin signaling increased the expression of the downstream target genes c-Myc and β-catenin to increase ovarian cancer cell proliferation and the proportion of S-phase cells throughout the cell cycle and reincreased platinum sensitivity. Meanwhile, the aforementioned studies also found that TMEM88 overexpression induced cell dormancy to help it evade the lethal effects of chemotherapy and trigger recurrent tumors. These studies suggest that TMEM88 may have an important regulatory role in the process of ovarian cancer resistance and may also be used to identify epigenetic modifiers associated with platinum resistance, which may provide new clues and insights for predicting ovarian cancer recurrence and overall cancer patient survival.

### TMEM88 in Breast Cancer

Authoritative research estimates that breast cancer has become the most prevalent cancer type among female cancer patients in the United States, and death also ranks second among female cancer-related death types, with a true proportion of 15% in 2022 (33). According to the latest data released by the National Cancer Center, the incidence of breast cancer in China is 7.11%, and the mortality rate is 2.81%, ranking fifth and seventh, respectively (34). Based on the above statistical results, breast cancer is a serious public health issue in current society and needs to be given great attention. Although great progress has been made in clinical treatment strategies, including surgery, chemotherapy, radiotherapy and hormone therapy, the long-term clinical prognosis and the survival rate of patients are still not satisfactory (35, 36). Therefore, it has become an urgent problem to be solved in breast cancer research to explore new therapeutic targets and develop effective intervention strategies. During the process of exploring new targets for breast cancer, Yu et al. conducted a study that first performed a correlation analysis between 139 breast cancer tissues and normal breast tissues. The results showed that the overall expression level of TMEM88 in breast cancer tissues (71.22%, 99/139) was significantly higher than that in normal tissue (11.4%, 4/35; *P* < 0.001), and the specific expression differences were not the same. Specifically, expression and localization analysis revealed that TMEM88 was moderately elevated in breast cancer *in situ* and highly elevated in invasive breast ductal carcinoma compared with negative or low expression in normal breast tissue, which suggests that the expression of TMEM88 has potential as a marker for breast cancer classification. In addition, the study also found that the cytoplasmic localization of TMEM88 was positively correlated with advanced TNM stage (*P* = 0.038) and lymph node metastasis (*P* = 0.01), while nuclear localization was inversely related to lymph node metastasis (*P* = 0.046). These results indicate that TMEM88 plays different functions depending on different subcellular localizations in the development of breast cancer (cytoplasmic TMEM88 promotes tumors, while nuclear TMEM88 suppresses tumors), suggesting that TMEM88 has important research value and needs to be considered (30). When using breast cancer cells (MDA-MB-231 and MCF-7) as the object to explore the mechanism, Yu et al. also found that TMEM88 and Dvl colocalized in the cytoplasm and TMEM88 can interact with Dvl to promote the expression of Snail protein and inhibit the expression of Zo-1 and Occludin, thereby reducing the invasion and metastasis of breast cancer cells. The above results show that the expression level of TMEM88 has certain differences in different types of breast cancer, and the cytoplasmic level and nuclear expression of TMEM88 have obvious tissue distribution specificity, which may facilitate TMEM88 becoming a promising therapeutic target for the treatment of breast cancer; however, the specific mechanism and intervention potential need further research to clarify.

### TMEM88 in Lung Cancer

Lung cancer has become the malignant tumor with the greatest threat to human health and life worldwide due to its higher incidence and mortality than other malignant tumors, accounting for 11.6% of cancer patients and 18.4% of total cancer deaths (37, 38). Among all lung cancer patients, NSCLC accounts for approximately 80%-85% of the total population. Although surgical resection is an effective treatment for early-stage lung cancer, the 5-year survival rate is still unsatisfactory (approximately 30-60%) (39, 40). What is even more regrettable is that most patients are already in the middle stage when they are diagnosed, most are even in the advanced stage, and they have lost the opportunity for surgical treatment, which accounts for one of the main reasons for the poor prognosis and high mortality of lung cancer patients (41). Therefore, exploring new targets and researching new treatment methods are urgently needed and will also provide a large number of theoretical and research foundations for the development of clinical treatments for lung cancer. In a study of 214 cases of NSCLC, Zhang et al. found that the expression of TMEM88 in the adjacent tissue was negative or weakly positive compared with the tissue of NSCLC patients by immunohistochemical analysis. The correlation analysis results of clinicopathological characteristics showed that high expression of TMEM88 in the cytoplasm was directly correlated with the tissue differentiation, lymph node metastasis and tumor stage of NSCLC patients (*P* < 0.001, *P* = 0.032 and *P* = 0.012), but there was no obvious correlation with sex, age, or histological type (*P* = 1, *P* = 0.884 and *P* = 1). Kaplan-Meier survival analysis showed that the overall survival of lung cancer patients (38.8 ± 4.83 months) with high TMEM88 expression was significantly lower than that of patients (58.64 ± 4.24 months) with low TMEM88 expression (26). This study preliminarily demonstrated the potential role and promising research value of TMEM88 in the development and exploration of NSCLC, but the specific pathogenesis needs to be further clarified. Subsequently, Zhang et al. used Western blotting to analyze the changes in TMEM88 in 40 fresh NSCLC specimens and found that TMEM88 expression was higher than that in normal lung tissue, which is also consistent with previous findings. Immunofluorescence staining of 7 types of lung cancer cells (A549, LTE, SPC, H292, H1299, BE1 and LH7 cells) found that the content of TMEM88 was significantly higher than that in human bronchial epithelial cells (HBE), while TMEM88 was found in the other two lung cancer cell types (LK2 and H460 cells) and was interestingly lower than that in HBE cells, and TMEM88 was predominantly located on the cell membrane in all types of lung cancer cells studied. Taking LK2 cells as the research object to study the effect of TMEM88, it was found that overexpression of TMEM88 can inhibit the excessive proliferation, invasion and migration of LK2 cells, thus preventing the growth of transplanted tumors in nude mice (26). Another study analyzed the NSCLC tissues and adjacent normal tissues of 12 patients and found that the degree of TMEM88 methylation in NSCLC tissues (82.2% ± 10.3%) was higher than that in normal tissues (65.9% ± 7.2%, *P* < 0.01), which was negatively correlated with overall survival (*P* = 0.021) (28). Subsequently, researchers used 5-aza-2’-deoxycytidine (DAC) to treat A549 and H1299 cells to further evaluate the functional alterations of TMEM88 methylation in lung cancer. The experimental results show that the degree of methylation of TMEM88 decreases while the expression level of TMEM88 increases after treatment with DAC. Scratch experiments, migration experiments and cell cycle experiments have found that TMEM88 can potently inhibit abnormal lung cancer cell proliferation, invasion and migration (26, 28), indicating that TMEM88 can exert a tumor suppressor effect and can be considered a candidate therapeutic target for the prognosis and cure of lung cancer, opening up a new avenue for the prevention of lung cancer.

### TMEM88 in Thyroid Cancer

Thyroid cancer (TC) has become one of the most common malignancies of the endocrine system over the past few decades, with a steady increase in global morbidity and mortality and a strong female predominance, which is well validated by the finding that the incidence in women in most populations is approximately three times that in men (42). Since 2003, the incidence of thyroid cancer in China has suddenly started to rise rapidly and has maintained an annual percentage change of approximately 14.51 from 2003 to 2007, while urban women have witnessed a faster rate of change, and by 2012, thyroid cancer had become one of the ten most common cancers (43–45). Meanwhile, one study also predicts that thyroid cancer may become the fourth most common malignant tumor in the world by 2030 (46). From a pathological point of view, thyroid cancer is mainly divided into papillary carcinoma, follicular carcinoma, anaplastic carcinoma and medullary carcinoma, among which papillary carcinoma is the most common (47). Most papillary thyroid carcinomas have a good prognosis, but local recurrence or distant metastasis after treatment is also common, and some patients die due to disease progression. Many studies have shown that various factors, such as radiation exposure, environmental and industrial pollution, and family inheritance, may promote the occurrence and development of thyroid cancer, but its pathogenesis is still unclear (43, 48). Therefore, exploring the pathogenesis of thyroid cancer and finding potential targets will provide an important basis for the preclinical study and clinical treatment of thyroid cancer. In one study, the researchers found that TMEM88 was significantly reduced in thyroid cancer by analyzing the gene expression profile interactive analysis database (25). In addition, Geng et al. also showed that the expression levels of TMEM88 were significantly decreased in 8 thyroid cancer patient specimens as well as in 4 thyroid cancer cell lines, BCPAP, TPC1, K1 and NPA87. Various experimental methods, such as Western blotting, qRT-PCR, cell counting kit-8 assay and colony formation experiments, found that restoration of TMEM88 by vector transfection can markedly suppress the proliferation, colony formation and invasion ability of thyroid cancer cells. In contrast, depletion of TMEM88 can accelerate the proliferation and invasion ability of thyroid cancer cells. The results of tumor formation experiments in nude mice also suggested that TMEM88 overexpression can significantly inhibit the growth of thyroid cancer, which is associated with the downregulation of active β-catenin expression. The above results show that TMEM88 exerts a nonnegligible role in the occurrence and development of thyroid cancer, and in-depth research on the mechanism of TMEM88 will offer new targets and ideas for the research and prevention of thyroid cancer.

### TMEM88 in Bladder Cancer

An authoritative analysis found that bladder cancer, the fifth most common cancer in Western countries, has an increasing incidence with age and is found in the highest proportion in individuals over the age of 65 (49). In China, the incidence of bladder cancer ranks second among male genitourinary malignancies and 7th among all malignant tumors (50). Clinical studies have shown that bladder cancer is a highly malignant tumor that is prone to recurrence and progresses rapidly, especially muscle-invasive BC (MIBC), which often recurs after the first resection and has a poor prognosis (51, 52). Therefore, it is particularly urgent to explore the possible pathogenesis and potential therapeutic targets of bladder cancer. In one study, research found that TMEM88 is closely relevant to the pathogenesis and development of bladder cancer, and inhibition of the TMEM88/Wnt axis can significantly affect abnormal bladder cancer cell proliferation and the regulation of the cell cycle (29), which suggests that targeting TMEM88 for bladder cancer research will be a new strategy and direction. Moreover, the study conducted bioinformatic analysis of the GEPIA2 and ENCORI databases and found that the expression of TMEM88 in bladder cancer tissues was significantly reduced compared to that in normal tissues. In the detection of 6 bladder cancer patient tissues, it was found that the expression of TMEM88 in patient tissues indeed showed a downward trend compared with adjacent normal tissues. *In vitro*, depletion of TMEM88 in bladder cancer cells, such as UM-UC-3 and T24 cells, can enhance their invasive and proliferative capacity, while restoring TMEM88 levels can reverse these effects. In *in vivo* experiments in nude mice, overexpression of TMEM88 exhibited a certain inhibitory action on bladder cancer cell growth and tumor formation (53). The above results show that TMEM88 is most likely to become the specific target and that targeting TMEM88 may effectively treat bladder cancer.

### Possible Mechanism of TMEM88 in Malignant Tumors

The relatively conserved Wnt signaling pathway is one of the major factors regulating development across the animal kingdom and is a key driver of stem cells in most types of tissues (54, 55). While regulating embryonic development and maintaining tissue homeostasis, it mediates downstream signals to participate in various biological/pathological processes and the genesis and development of cancer (53, 56, 57). During the research process of TMEM88, studies have pointed out that the C-terminal tripeptide Val-Trp-Val sequence of a subtype of TMEM88 CRA-a can bind to the PDZ domain of the scrambled protein and prohibit the Wnt/β-catenin signaling pathway, which exerts a nonnegligible effect in regulating tumor cell proliferation, metastasis and host antitumor immunity (22, 27, 58). For example, it can be coexpressed in the cytoplasm with scattered proteins in NSCLC and breast cancer to elevate the levels of Snail and thereby promote tumor progression. Analysis of the transcriptome information of ovarian cancer patients in the TCGA database shows that TMEM88 is closely correlated with the mRNA expression levels of c-Myc and β-catenin mRNA (*P* = 0.01252 and 0.0128) (19). In addition, Geng et al. found that the overexpression of TMEM88 significantly reduced the transcriptional activity of TCF/LEF and inhibited the expression of the downstream target genes c-Myc and cyclin D1 of the Wnt/β-catenin signaling pathway (25). Furthermore, reactivation of Wnt/β-catenin signaling by transfection of the pENTRN90-β-catenin vector partially reversed the inhibitory effect of TMEM88 on the proliferation and invasion of thyroid cancer cells, indicating that TMEM88 exerts an anti-thyroid cancer effect in the presence of Wnt/β-catenin protein signaling. A study in bladder cancer found that overexpression of TMEM88 also inhibited the activation of the Wnt/β-catenin signaling pathway by reducing the phosphorylation level of GSK-3β (Ser9 site) (29). However, in a study of triple-negative breast cancer, transfection of MCF-7 and MDA-MB-231 cells to overexpress or silence TMEM88 did not affect the activity of the canonical Wnt signaling pathway or the expression of corresponding target genes such as MMP-7, c-Myc and cyclin D1 (30). Taken together, the above studies suggest that TMEM88, as an important linker in the genesis and development of various cancer diseases, can function by regulating different downstream oncogenic signals and effector molecules. It transmits upstream signals and regulates downstream signal transduction, such as Wnt signaling pathways, to interfere with the occurrence and progression of tumors and then has an anticancer effect. Based on this, TMEM88 should be considered a novel and important research target in cancer research, which needs to be deeply explored for its potential.

## Targeting TMEM88 Potential for Treating Malignant Tumors

With the deepening of people’s understanding of tumor research and clinical, molecular targeted therapy has also received increasing attention and aroused the interest of researchers. At present, the most common molecular targeted therapeutic drugs mainly include small molecular epidermal kinase inhibitors, such as gefitinib (AstraZeneca, UK) (59); anti-EGFR monoclonal antibodies, such as cetuximab (Merck & Leone Pharmaceutical Co, Germany) (60); anti-HER-2 monoclonal antibodies, such as trastuzumab (Roche Group, Switzerland) (61); Bcr-Abl tyrosine kinase inhibitors, such as imatinib (Novartis, Switzerland) (62); anti-CD20 monoclonal antibodies, such as rituximab (Roche Group, Switzerland) (63); and vascular endothelial growth factor receptor inhibitors, such as bevacizumab (Roche Group, Switzerland) (64). When the above drugs exert antitumor effects, they can significantly reduce the toxicity to normal cells, which provides a new direction for molecular targeted therapy of tumors. However, due to the heterogeneity of cancers, the current targeted therapeutic drugs benefit only a portion of cancer patients, which forces us to explore a broader spectrum of malignant tumor therapeutic targets and develop promising therapeutic drugs to address the urgent issue in tumor research and current clinical practice.

To date, a variety of transmembrane proteins have become powerful targets for drug development and have even entered clinical trials and clinical applications. As a representative, the G protein coupled receptor (GPCR) family is the largest transmembrane protein family in humans and is also an important target of many drugs (65). Currently, 475 drugs targeting GPCR have been approved by the FDA, accounting for 34% of all FDA approved drugs. Meanwhile, 321 drugs targeting GPCRs are in clinical studies (66, 67). The above drugs are mainly distributed in small molecule drugs, polypeptide drugs, monoclonal antibodies, and recombinant proteins. Four-transmembrane proteins, CD20 and Claudin 18.2, are important targets for disease treatment and drug development. CD20 is a transmembrane phosphorin located on the surface of B lymphocytes, mainly in the preceding B-cell to mature B-cell stage (68). Currently, the antitumor drug antibody and inflammatory immune regulatory antibody drugs that target CD20 have entered the fast lane developed by the product (69). Claudin18.2 is the most important member of the Claudin transmembrane protein family. In the normal physiological state, Claudin18.2 protein is only expressed on the surface of human gastric epithelial shorthearter cells. However, in the pathological state, Claudin18.2 protein is highly expressed in gastric cancer, esophageal cancer, pancreatic cancer, and other solid tissues (15, 70–72). The significant differences and high tissue specificity make Claudin 18.2 an ideal target for solid tumor immunotherapy(2016). Currently, it has been close to 20 drugs targeting Claudin18.2 in the clinical phase, such as zolbetuximab (Phase III: NCT03653507, Astellas Pharma Inc., Japan), TST001 (Phase I: NCT04495296, MabSpace Biosciences, China) and AMG910 (Phase I: NCT04260191, Amgen Inc., USA), and these drugs are primarily concentrated on monoclonal antibody and bispecific antibody, antibody-drug conjugate (ADC), and chimeric antigen receptor T-cell immunotherapy (CAR-T) (72–75). The abovementioned drug development of transmembrane proteins has laid a solid foundation for the research and exploration of targeted TMEM88 to treat malignant tumors. Meanwhile, we should be more aware of the current gaps in the development of drugs targeting TMEM88 for the treatment of tumors: 1. Insufficient analysis of the three-dimensional and crystal structure of the TMEM88 protein makes the structural information of the protein lacking. 2. Research on the full length of the TMEM88 protein polypeptide and the recognition epitope is insufficient, and information on the active site and affinity is lacking. We believe that the resolution of these problems will greatly promote the development of drugs targeting TMEM88.

However, due to the surface area of the transmembrane protein structure, its expression level is difficult to meet the requirements. Furthermore, it is difficult for us to achieve high-purity multitransmembrane proteins with natural conformations and activity. These difficulties make targeted TMEM88 antitumor drug development difficult. Based on this, various strategies can be considered to increase the development of antitumor drugs targeting TMEM88: 1. Novel protein purification, isolation and characterization systems and techniques need to be applied; 2. Artificial intelligence and computer simulation need to be more invested; 3. Drug diversity needs to be constantly tried, such as antibody drugs, nanodrugs, small-molecule drugs, traditional Chinese medicines and proteolysis targeting-chimeras (PROTAC). In any case, as a two-transmembrane protein, TMEM88 has significant differences and specificities between multiple malignant tumor and adjacent tissues and tumor and normal cells, which makes TMEM88 a potential therapeutic target through different strategies during tumor treatment.

## Prospective and Conclusions

In recent years, tumors have been the main cause of human death, and the occurrence and development of many kinds of tumors are closely related to the imbalance of signal transduction in the body (76). Therefore, it is extremely important to clarify the signal transduction mechanism and find potential new tumor therapeutic targets. In the current literature, TMEM88 is considered to play an important role in the development of breast cancer, lung cancer, thyroid cancer and other tumors by inhibiting Wnt signal transduction and participates in the occurrence and development of tumors, but it needs to be pointed out that TMEM88 research is not deep enough, and many issues remain to be studied. Specifically, TMEM88 has different expression levels in different tumors and has tissue specificity, but what are the specific characteristics; when there are multiple influencing factors, will the role of TMEM88 in malignant tumors be different? TMEM88 methylation affects tumor biology However, what is the specific mechanism? In the process of cancer development, TMEM88 not only regulates the Wnt/β-catenin signaling pathway and participates in it but also affects other signal transduction processes. Therefore, more basic research needs to be carried out to further confirm the specific role of TMEM88 in tumor development, improve its credibility as a potential therapeutic target, and prove its potential to transform from basic research to clinical application in tumor treatment. In any case, the results of multiple studies have shown that TMEM88 may be a tumor diagnosis and prognostic indicator, which will provide new clues and directions for the diagnosis and treatment of clinical malignant tumors. We look forward to using advanced technologies for in-depth research, such as microarray analysis, tissue sample sequencing, high-throughput DNA methylation analysis and other new technologies, revealing the function of TMEM88 regulation in more malignant tumors and elucidating the pathogenesis of TMEM88 in tumors. This will further promote the progress of the diagnosis and treatment of malignant tumors.

## Author Contributions

HZ, W-JN, and MC designed the “idea”; MC and W-JN wrote the manuscript; Y-HW and J-JW collected the information; W-JN and HZ revised the manuscript. All authors read and approved the final manuscript.

## Funding

This work was supported by the Science Foundation of Anhui Provincial Cancer Hospital (No. 2020YJQN008), the National Natural Science Foundation of China (No. 81803602), the Natural Science Foundation of Anhui Province (No. 1708085QH207), and the Fundamental Research Funds for the Central Universities (No. WK9110000018). Key Project of the Clinical Research Fund of Anhui University of Chinese Medicine (No. 2021efylc03).

## Conflict of Interest

The authors declare that the research was conducted in the absence of any commercial or financial relationships that could be construed as a potential conflict of interest.

## Publisher’s Note

All claims expressed in this article are solely those of the authors and do not necessarily represent those of their affiliated organizations, or those of the publisher, the editors and the reviewers. Any product that may be evaluated in this article, or claim that may be made by its manufacturer, is not guaranteed or endorsed by the publisher.
